# 

**Published:** 1899-08-05

**Authors:** 


### NOTICE TO CORRESPONDENTS.

All MS., Letters, Books for Review, and other matters intended for the Editor should be addressed The Editor, "The Hospital," The Hospital Building, 28 & 29, Southampton Street, Strand, W.C.

The Editor cannot undertake to return rejected MS., even when accompanied by stamped directed envelope.

**Notice to Advertisers and Subscribers.**

All Advertisements, Orders for Copies of The Hospital, and Business Communications should be addressed to **THE MANAGER (Not to the Editor),** The Hospital, The Hospital Building, 28 & 29, Southampton Street, Strand, London, W.C.

The Cost of Subscription, post free, is as follows:—Per week, 2½d.; per quarter, 2s. 8d.; per half-year, 5s. 3d.; per annum, 10s. 6d. Foreign:—Per annum, 15s. 2d., payable, in all cases, in advance.

**NOTICE.—Vol. XXV. of "The Hospital" (October, 1898, to March, 1899), in handsome cloth binding, is now on sale at the Office of this Journal, price 7s. 6d. Cases for binding the Numbers contained in this Volume 1s. 6d. Index to Vol. XXV., 2½d., post free.**

**The Monthly Part for August is now ready. Price 6d., by post 8d.**

### BOOKS RECEIVED.

Macmillan and Co.
"A System of Medicine by Many Writers." Edited by Thomas Clifford Allbutt, M.D., F.R.C.P., F.R.S.

The Scientific Press.
"Medical Gymnastics, including the Schott (Nauheim) Movements." By Alex. V. Grafstrom, B.Sc., M.D.

Sampson Low, Marston, and Co.
"Scheveningen." By Dr. W. Francken.

Great Eastern Railway Company.
"Tourist Guide to the Continent." Edited by Percy Lindley.

### The Student of Medicine.

### APPOINTMENTS.

J. E. Baker, M.B., B.S.Durh., appointed House Surgeon to the North Shields and Tynemouth Dispensary. J. Berry, B.S., F.R.C.S., appointed Surgeon to the North London Hospital for Consumption and Diseases of the Chest. A. W. Brown, M.A., B.A., D.Sc.Aberd., appointed an additional Examiner in Botany at the University of Aberdeen. M. Connon, M.B., C.M., D.P.H., M.O.H. Montrose, appointed Police Surgeon to the Burgh. A. Duncan, M.D., B.S.Lond., F.R.C.S. appointed a Physician to In-patients, Branch Hospital Seamen's Hospital Society. W. T. Freeman, M.D.Durh., F.R.C.S.Eng., appointed Surgeon in Charge of Department for Diseases of Skin at the Reading Dispensary. H. E. Harris, M.B., F.R.C.S.Eng., appointed Surgeon to the Out-patient Department of the Bristol Royal Hospital for Sick Children and Women. F. W. N. Haultain, M.B.Edin., appointed an additional Examiner at the University of Aberdeen. A. C. Houston, M.D., D.Sc., appointed Lecturer in Bacteriology at the Bedford College, London. C. E. Lansdown, M.R.C.S., L.R.C.P.Lond., appointed Surgeon to the Hospital for Sick Children, Cheltenham. C. H. Leaf, M.A., M.B.Cantab., F.R.C.S.Eng., appointed Assistant Surgeon to the Cancer Hospital, Fulham Road, Brompton. D. W. Maclagan, M.B., Ch.B., appointed House Surgeon to the Edinburgh Royal Maternity and Simpson Memorial Hospital. J. B. McLaren, M.A., M.B., B.Ch., appointed Medical Officer to the Workhouse of the Salford Union. F. H. Rose, M.R.C.S.Eng., L.R.C.P.Lond., appointed Anæsthetist to the Bristol Royal Hospital for Sick Children and Women. F. O. Simpson, L.R.C.P.Lond., M.R.C.S.Eng., appointed Senior Assistant Medical Officer to the County Asylum, Rainhill, near Liverpool. W. H. Symons, M.D., D.P.H., F.I.C., appointed Medical Officer of Health for the City and County of Bath. A. F. Voelcker, M.D.Lond., appointed an additional Examiner in Medicine at the University of Aberdeen. M. Wedderburn, M.B., Ch.B., appointed House Surgeon to the Edinburgh Royal Maternity and Simpson Memorial Hospital.

